# The Incidence and Predictive Factors in the Development of Acute Hepatitis in Patients with Leukemia

**DOI:** 10.5005/jp-journals-10018-1254

**Published:** 2018-05-01

**Authors:** Muhsin Kaya, Recai Akdogan, Feyzullah Uçmak, Mehmet O Ayyildiz, Abdullah Karakus, Muhammet A Kaplan

**Affiliations:** 1 Department of Gastroenterology, Dicle University School of Medicine, Diyarbakir, Turkey; 2 Department of Internal Medicine, Dicle University School of Medicine, Diyarbakir, Turkey; 3 Department of Hematology, Dicle University School of Medicine, Diyarbakir, Turkey; 4 Department of Oncology, Dicle University School of Medicine, Diyarbakir, Turkey

**Keywords:** Acute hepatitis, Complications, Leukemia.

## Abstract

**Introduction:**

Liver involvement is common in hematological malignancies, but the incidence and pattern of liver injury vary among the different types. The aims of our study were to determine the incidence and clinical course of acute hepatitis and the important factors for its development in patients with leukemia after chemotherapy.

**Materials and methods:**

All patients with the diagnosis of leukemia who were treated at the Department of Hematology between January 2008 and January 2013 were included in the study. A detailed medical history, clinical and laboratory findings, treatment modalities, complications, and clinical course were recorded retrospectively.

**Results:**

A total of 124 patients (64 females) with the diagnosis of leukemia were included in the study. The mean age was 45.2 years (16-89 years) and mean follow-up time was 29.7 months (0.25-192 months). A total of 43 (34.6%) patients had acute hepatitis after chemotherapy. Pattern of liver injury was hepatocellular in 31 patients, cholestasis in 2, and mix in 10 patients. Age (p = 0.001), hepatitis B surface antigen (HBsAg, p = 0.007), acute leukemia (p < 0.001), positive blood culture (p = 0.004), the amount of transfused red blood cell (p = 0.001), and amount of transfused platelets (p = 0.002) were significantly different under univariate analysis between the acute hepatitis group and the nonacute hepatitis group. Under multivariate analysis, only acute lymphoblastic leukemia (ALL) was identified as independent predictive factor for development of acute hepatitis after starting chemotherapy.

**Conclusion:**

Acute and self-limited hepatitis develops in the substantial proportion of patients with leukemia. The most important factor for development of acute hepatitis is the type of leukemia.

**How to cite this article:** Kaya M, Akdogan R, Uçmak F, Ayyildiz MO, Karakus A, Kaplan MA. The Incidence and Predictive Factors in the Development of Acute Hepatitis in Patients with Leukemia. Euroasian J Hepato-Gastroenterol 2018;8(1):31-37.

## INTRODUCTION

Gastrointestinal manifestations of leukemia occur in up to 25% of patients at autopsy, generally during relapse and more common in acute than chronic leukemia. Its presence varies with the type of leukemia and has been decreased over time due to improved chemotherapy. Gross leukemic lesions are most common in the stomach, ileum, and proximal colon and include nodules, plaques, diffuse infiltrates, polyps, and a convoluted brainlike appearance of the mucosal folds. Patients with leukemic infiltrates are usually asymptomatic or have vague, nonspecific complaints. They may present with abdominal pain, diarrhea, or bleeding.^1^ Patients with acute or chronic leukemia may present with cholecystitislike symptoms and gallbladder wall infiltration.^2,3^ Pancreatitis is rare and may be due to L-asparaginase, even 10 weeks after stopping therapy.^1^ Infiltration of lymphoreticular organs, mainly spleen, liver, and lymph nodes, occurs in many patients with leukemia and is more prominent in chronic than acute disease.^4^ Liver involvement is common in hematological malignancies, but the incidence and pattern of infiltration vary among the different types of disease. Liver infiltration has been reported in 80 to 100% of chronic leukemia and in 60 to 70% of acute leukemia. Diffuse, nondestructive infiltration in acute myelocytic leukemia (AML), both the portal and sinusoidal infiltration in chronic myelocytic leukemia (CML), mainly sinusoidal infiltration in ALL have been reported.^5^

Sinusoidal obstructing syndrome (SOS), previously known as hepatic veno-occlusive disease, is another cause of hepatic dysfunction in patients with leukemia. It occurs most commonly as a complication of myeloablative regimens. High-dose chemotherapy alone or combined with total body irradiation, mainly cyclophosphamide in combination with either busulfan or total body irradiation or regimens that include multiple alkylating agents, are commonly implicated.^1^

Imatinib mesylate,^6,7^ hydroxyurea,^8^ L-asparaginase,^9^ arsenic trioxide,^10^ which are used in the treatment of leukemia, can cause hepatitis. The clinical presentation of hepatic dysfunction may range from asymptomatic elevation of aspartate aminotransferase (AST) and alanine aminotransferase (ALT) to fulminant hepatic failure. Liver injury can be described as cholestatic, hepatocellular, or mixed in nature.^11^

Chronic hepatitis caused by hepatitis E,^12^ acute hepatitis caused by hepatitis A^13^ and hepatitis B^14,15^ have been reported in patients with leukemia. Reactivation of hepatitis B infection is an important cause of acute hepatitis in patients with hematological malignancy. Syncytial giant cell hepatitis,^16^ Budd-Chiari syndrome,^1^ ischemic hepatitis,^17^ sepsis,^18^ and cholestasis induced by total parenteral nutrition^19^ are some causes of hepatic dysfunction in patients with leukemia.

The aims of our study were to determine: (1) The incidence of acute hepatitis (2) the important factors in the development of acute hepatitis in patients with leukemia after starting chemotherapy.

## MATERIALS AND METHODS

### Patients

All patients with the diagnosis of leukemia who were treated at the Department of Hematology, Dicle University School of Medicine, Turkey, between January 2008 and January 2013 were included in our study. A detailed medical history was documented retrospectively for all patients enrolled in the study. These include age, gender, family and personal history of liver disease, alcohol consumption, and details of any significant systemic diseases. Additionally, the amount of transfused red cell packed, fresh frozen plasma and platelet, the results of blood and urine culture during therapy have been recorded. In case of the development of liver function tests abnormality, radiological investigations, including abdominal ultrasound and/or computerized tomography, were performed for excluding vascular and space occupying liver diseases. Lamivudine 100 mg/day was commenced immediately in case of positivity of HBsAg in all patients and continued until death or last follow-up. All patients have been followed from diagnosis until death or last visit date.

The study was approved by Ethics Committee of Dicle University, School of Medicine, Diyarbakir, Turkey.

### Chemotherapy Regimens

Table 1 shows different chemotherapeutic agent for different type of each leukemia. Patients were also administered several antibiotics in case of suspected or documented infection.

### Liver Function Tests

Serum ALT, AST, gamma-glutamyl transpeptidase (GGT), alkaline phosphatase (ALP), bilirubin, albumin, prothrombin time were measured on a 24-factor automated chemical analyzer using standard reagent. The normal range was 0 to 40 IU/L for AST and ALT, 0 to 57 IU/L for GGT, 41 to 117 IU/L for ALP, 0 to 1.4 mg/dL for bilirubin, 3.5 to 5.5 g/dL for albumin, <13 seconds for prothrombin time. In patients with diagnosis of acute leukemia, during the course of chemotherapy, the tests were carried out routinely before each chemotherapy cycle, at 2-day intervals during the first 2 weeks after each chemotherapy cycle, and then every 2 to 4 weeks interval during follow-up. In patients with chronic lymphocytic leukemia (CLL), the tests were carried out routinely before each chemotherapy cycle and then every 2 to 4 weeks interval during follow-up. In patients with CML, the tests were carried out routinely every 2 to 4 weeks interval during follow-up. In case of development of acute hepatitis, liver function test was measured in shorter intervals. We did not perform baseline or follow-up liver biopsy in all cases.

**Table 1:** **Table 1:** Chemotherapeutic agents given for different leukemia

*Diagnosis*		*Chemotherapeutic agent*	
Acute		Remission induction: cytosine arabinoside,	
myelocytic		idarubicin	
leukemia		Consolidation: high-dose cytosine arabinoside	
		Relapse or refractor AML: fludarabine,	
		cytosine arabinoside, idarubicin or	
		mitoxantrone, cytosine arabinoside, etoposide	
Acute		Remission induction: cyclophosphamide,	
lymphoblastic		vincristine, dexamethasone, adriamycin or	
leukemia		methotrexate, folinic acid, cytosine arabinoside	
		Maintenance treatment: methyl prednisone,	
		vincristine, methotrexate, 6-mercaptopurine	
Chronic		Imatinib mesylate, hydroxyurea	
myelocytic			
leukemia			
Chronic		Chlorambucil or rituximab, fludarabine, cyclo-	
lymphocytic		phosphamide or rituximab, cyclophosphamide,	
leukemia		vincristine, methyl prednisone	

### Serological Tests

Hepatitis B surface antigen, hepatitis B surface antibody (anti-HBs), hepatitis B e antigen (HBeAg), hepatitis B e antibody (anti-HBe), hepatitis B core antibody [anti-HBc immunoglobulin (Ig)M, IgG], hepatitis Delta antibody (anti-D), hepatitis A virus antibody (anti-HAV IgM, IgG), cytomegalovirus (CMV) antibody, Epstein-Barr virus (EBV) antibody, and human immunodeficiency virus (HIV) antibody were all assayed with second-generation enzyme-linked immunosorbent assay (ELISA; Abbott Laboratories, North Chicago, Illinois, USA). Hepatitis C virus antibody (anti-HCV) was assayed using a second-generation ELISA. Serum hepatitis B virus (HBV)-DNA was tested by dot-blot (liquid) hybridization on all serum samples available from all the patients who were HBsAg positive. HBsAg, anti-HBsAg, and anti-HCV were assessed before chemotherapy in all patients. HBeAg, anti-HBe, anti-HBcIgM, anti-HBcIgG, and anti-D were assessed in case of positivity of HBsAg. Remaining viral marker was assessed in case of development of acute hepatitis.

### Definitions

Acute hepatitis was defined as an elevation of serum ALT level to more than three times the upper limit of normal (ULN, <40 IU/L) in the context of a doubling of patients’ baseline value on two consecutive determinations at least 5 days apart followed by recovery of liver function within 6 months. Chronic active hepatitis was defined as an ALT elevation lasting for more than 6 months. Pattern of liver injury was assessed using R value, where R = (ALT/ULN)/ (ALP/ULN). Hepatocellular pattern was defined for R-value ≥5, mixed pattern for R-value >2 and <5, cholestasis pattern for R-value ≤2. Hepatitis B virus reactivation was defined by the reappearance or an increase of >1 log_10_ (or >20,000 IU/mL) of serum HBV-DNA in patients with previously resolved (occult infection), active, or inactive HBV infection in the absence of laboratory evidence of acute infection with hepatitis A, C, and delta viruses. Lamivudine 100 mg/day was commenced immediately in case of positivity of HBsAg in all patients and continued until death or last follow-up.

### Statistical Analysis

The Mann-Whitney U-test for continuous variables and the chi-square test for categorical variables were used in our analysis as appropriate. Multiple logistic regression analysis was performed to identify the independent factors that were significantly associated with the development of acute hepatitis. Candidate variables with a p-value of <0.05 on univariate analysis were entered into the regression analysis. A p-value of <0.05 was considered significant. Statistical analysis was performed using Statistical Package for the Social Sciences version 10 (SPSS, Inc., Chicago, Illinois).

## RESULTS

### Baseline Characteristics of Patients

A total of 124 patients (64 females, 60 males) with the diagnosis of leukemia were included in the study. The mean age was 45.2 (16-89) years and mean follow-up time was 29.7 (0.25-192) months. Seventeen patients have diabetes mellitus. None of the patients had a history of alcohol consumption. There were AML in 63 (50.8%) patients, ALL in 16 (13.2%), CML in 21 (16.9%), and CLL in 24 (19.3%) patients.

Table 2 shows the baseline characteristics of patients with and without acute hepatitis after starting chemotherapy. A total of 4 patients had acute hepatitis before commencement of chemotherapy. A total of 43 (34.6%) patients, including 4 patients who initially have acute hepatitis, had experienced acute hepatitis after starting chemotherapy. Pattern of liver injury was hepatocellular in 31 (72%) patients, cholestasis in 2 (4.6%), and mix in 10 (23.2%) patients.

There was acute hepatitis in 21 out of 63 (33.3%) patients with acute AML, 14 out of 16 (87.5%) patients with ALL, 2 out of 21 (9.5%) patients with CML, and in 6 out of 24 (25%) patients with CLL. Acute hepatitis was developed more frequently in patients with acute leukemia compared with chronic leukemia (44.3 *vs* 17.7%, p < 0.001) and more frequently in patients with ALL compared with AML (87.5 *vs* 33.3%, p < 0.001). There was no significant difference between patients with and without acute hepatitis regarding gender. Mean age of patients with acute hepatitis was significantly lower than age of patients without acute hepatitis (37.86 ± 2.64 *vs* 49.5 ± 2.03, p = 0.001).

**Table 2:** **Table 2:** Basic characteristics of patients with and without acute hepatitis

		*Patients with acute hapatitis*		*Patients withot acute hapatitis*			
*Parameter*		*(n = 43)*		*(n = 81)*		*p-value*	
Age (range)		37.86 ± 2.64		49.5 ± 2.03		0.001	
		(19-83)		(16-89)			
Gender (M/F)		24/19		36/45		0.155	
Positivity of HBsAg (n)		6		1		0.007	
AML		21		42		<0.001	
ALL		14		2			
CML		2		19			
CLL		6		18		NS	
Dead, n (%)		15 (34.8%)		16 (19.7%)		0.053	

NS: Not significant

### Biochemical Findings

Tables 3 and 4 show biochemical results of patients with and without acute hepatitis before and after starting chemotherapy. Nine of 124 (7.2%) patients, including 7 AML, 1 ALL, and 1 CML, had elevated liver enzyme level before starting chemotherapy. There was no significant difference between groups regarding biochemical results except serum peak ALT and peak GGT levels before chemotherapy. Serum peak ALT and peak GGT levels were significantly higher in patients with acute hepatitis compared with patients without acute hepatitis (40.9 ± 9.4 *vs* 17.5 ± 1.5 U/L, p < 0.001; 88.2 ± 31.4 *vs* 37.1 ± 4.1 U/L respectively). Serum ALT, AST, ALP, and GGT levels were significantly higher in patients with acute hepatitis compared with patients without acute hepatitis after starting chemotherapy. There was no significant difference between two groups regarding serum total bilirubin, albumin, and prothrombin time after starting chemotherapy. Liver enzymes were returned to normal level in 40 out of 43 patients in mean 61 ± 15 (6-180) days.

Liver enzyme elevation lasting for more than 6 months was observed in 3 out of 43 patients, including 2 patients with AML and 1 patient with CLL. All these patients had no viral infection and alive until last follow-up. These patients were considered to have chronic hepatitis.

**Table 3:** **Table 3:** Biochemical results of patients with and without acute hepatitis before starting chemotherapy

*Parameter*		*Patients with acute hepatitis*		*Patients without acute hepatitis*		*p-value*	
Peak ALT (U/L) (range)		40.9 ± 9.4 (6-319)		17.5 ± 1.5 (6-82)		<0.001	
Peak AST (U/L) (range)		51.4 ± 16.6 (9-520)		23.9 ± 1.7 (7-89)		0.087	
Peak ALP (U/L) (range)		109.8 ± 30.2 (12-1,286)		85.7 ± 6.1 (39-412)		0.994	
Peak GGT (U/L) (range)		88.2 ± 31.4 (9-1,220)		37.1 ± 4.1 (9-231)		0.030	
Peak total bilirubin (mg/dL)		0.91 ± 0.17 (0.2-7.8)		0.68 ± 0.04 (0.2-0.5)		0.368	
Lowest albumin (mg/dL)		3.4 ± 0.09 (1.9-4.8)		3.57 ± 0.07 (1.6-4.7)		0.181	
Peak prothrombin time (seconds) (range)		13.7 ± 0.3 (11.1-19.7)		14.12 ± 0.39 (11.3-39)		0.463	

**Table 4:** **Table 4:** Biochemical results of patients with and without acute hepatitis after starting chemotherapy

*Parameter*		*Patients with acute hepatitis*		*Patients without acute hepatitis*		*p-value*	
Peak ALT (U/L) (range)		274.4 ± 25.7 (71-735)		40.5 ± 3.3 (6-117)		<0.001	
Peak AST (U/L) (range)		263.1 ± 84.9 (36-3,530)		37.37 ± 2.5 (10-115)		<0.001	
Peak ALP (U/L) (range)		166 ± 25.1 (39-946)		99.9 ± 6.9 (45-390)		0.001	
Peak GGT (U/L) (range)		181.3 ± 30.8 (29-1,020)		61.29 ± 7.4 (10-342)		<0.001	
Peak total bilirubin (mg/dL)		1.73 ± 0.31 (0.4-10.9)		0.89 ± 0.14 (0.1-10.7)		0.001	
Lowest albumin level (g/dL)		3.26 ± 0.13 (1.3-4.7)		3.47 ± 0.08 (1.4-4.8)		0.154	
Prothrombin time (seconds)		15.11 ± 1.04 (11.6-43.9)		13.89 ± 0.43 (10.7-43.1)		0.383	

**Table 5:** **Table 5:** Clinical and laboratory features of patients with HBV infection

*Age/gender*		*Diagnosis*		*HBeAg*		*Anti-HBe*		*Initial HBV-DNA (IU/mL)*		*Initial liver enzyme*		*Acute hepatitis after chemotherapy*	
57/M		AML		Negative		Negative		1,000		Normal		Yes	
58/M		CLL		Positive		Negative		1,000,000		Normal		Yes	
61/M		CLL		Positive		Negative		641,000		Normal		Yes	
52/M		CLL		Positive		Positive		2,100		Normal		Yes	
29/M		ALL		Negative		Positive		1,000		Normal		Yes	
36/F		AML		Negative		Negative		100		Normal		Yes	
16/M		AML		Negative		Positive		4,500		Normal		No	

### Viral Serology

Table 5 shows clinical and laboratory features of patients with HBV infection. The HBsAg was positive in 7 (6 male) out of 124 (5.6%) patients before starting chemotherapy. These patients did not have any radiological features that can be compatible with chronic liver disease. Liver enzyme level was normal and HBV-DNA was positive in all patients before starting chemotherapy. These patients were accepted as chronic HBV infection. The positivity of HBsAg was significantly higher in patients with acute hepatitis compared with patients without acute hepatitis (13.9 *vs* 1.2% respectively; p = 0.007). Of patients with chronic HBV infection, 3 patients had AML, 3 CLL, and 1 ALL. None of the patients had anti-D antibody. Lamivudine was commenced before starting chemotherapy and continued until last follow-up in all patients. Acute hepatitis has developed in 6 out of 7 patients. These 6 patients had continued HBV-DNA positivity during follow-up. But, HBV-DNA did not increase after development of acute hepatitis compared with initial HBV-DNA level in all patients. Liver enzymes decreased to normal level after mean 35.5 (8-117) days. A 16-year-old male AML patient with positive HBV-DNA had not experienced acute hepatitis during follow-up. The positivity rate of HBsAg was significantly higher in patients with acute hepatitis compared with patients without acute hepatitis (p = 0.007). Anti-HCV antibody was negative in all patients with and without acute hepatitis. Anti-HAV IgM, CMV IgM, and EBV IgM antibody were negative in all patients with acute hepatitis.

### Amount of Transfused Blood Products and Results of Cultures

Table 6 shows the amount of blood products and results of cultures in patients with and without acute hepatitis. The number of transfused red blood cell packed and platelets was significantly higher in patients with acute hepatitis compared with patients without acute hepatitis (11.04 ± 1.4 *vs* 8.03 ± 2.5, p = 0.001; 8.7 ± 1.25 *vs* 5.67 ± 0.94, p = 0.002 respectively). There was no significant difference between two groups regarding the amount of transfused fresh frozen plasma.

Blood culture has been taken from 40 out of 43 (93%) patients with acute hepatitis and from 57 out of 81 (70%) patients without acute hepatitis during follow-up. Blood culture was positive in 22 out of 40 (55%) patients with acute hepatitis and in 15 out of 57 (26.3%) patients without acute hepatitis. The most commonly isolated bacteria from blood culture were Staphylococcus in both groups. The positivity rate of blood culture was significantly higher in patients with acute hepatitis compared with patients without acute hepatitis (55 *vs* 26.3%, p = 0.004). Urine culture has been taken from 38 out of 43 (88.3%) patients with acute hepatitis and from 57 out of 81 (70.3%) patients without acute hepatitis. The most commonly isolated bacteria from urine culture were *Escherichia coli* in both groups. There was no significant difference between two groups regarding positivity of urine culture.

### Clinical Course

We did not observe significant clinical and laboratory deterioration after starting chemotherapy in patients who had initial acute hepatitis. There was no acute liver failure in any patients. A total of 31 patients including 15 patients with acute hepatitis and 16 patients without acute hepatitis were died during follow-up (mean 29.7 ± 3.2) months. Mortality was not related to liver failure in all patients. There was no statistically significant difference between patients with and without acute hepatitis regarding mortality rate during follow-up.

### Independent Predictor for Development of Acute Hepatitis after Commencement of Chemotherapy

A multivariate logistic regression analysis was performed to determine the independent predictors that affected the development of acute hepatitis after commencement of chemotherapy using variables that were significant in the univariate analysis. Under multivariate analysis, only diagnosis of ALL was identified as independent factor for development of acute hepatitis (Table 7).

**Table 6:** **Table 6:** The amount of transfused blood products and the positivity of cultures in patients with and without acute hepatitis

*Parameter*		*Patients with acute hepatitis*		*Patients without acute hepatitis*		*p-value*	
Positivity of blood culture (n %)		22/40 (55%)		15/57 (26.3%)		0.004	
Positivity of urine culture (n %)		14%38 (36.8%)		15/57 (26.3%)		0.193	
Transfused red blood cells (units) (mean ± SD)		11.07 ± 1.4		6.27 ± 0.77		0.001	
Transfused platelet (units) (mean ± SD)		8.7 ± 1.25		5.67 ± 0.94		0.002	
Transfused fresh frozen plasma (units) (mean ± SD)		11.04 ± 4.35		8.03 ± 2.5		0.122	

**Table 7:** **Table 7:** Multivariate analysis of risk factors affecting the development of acute hepatitis after starting chemotherapy

		*Relative risk*		*95% confidence interval*		*p-value*	
Age		0.973		0.936-1.011		0.156	
Transfused red blood cell (units)		1.024		0.934-1.122		0.620	
Transfused platelet (units)		1.008		0.916-1.109		0.876	
Positivity of blood culture		2.170		0.671-7.021		0.196	
ALL		26.9		3.152		0.003	
Positivity of HBsAg		0.218		0.019-2.552		0.225	

## DISCUSSION

Gastrointestinal involvement is more common in acute than chronic leukemia and situated mainly in the mucosa and submucosa. Except for an occasional report, gastrointestinal involvement occurs when the leukemia is in relapse. Its presence varies according to the type of leukemia and has been decreasing over time due to improved chemotherapy.^1^ The liver can be involved to a variable extent, with mildly abnormal liver function tests being often the only abnormality without clinically evident liver disease. Rarely, fulminant liver failure may occur as a result of extensive replacement of hepatocytes with malignant cells, in particular in lymphoblastic leukemia. Characteristic histological findings in myeloid leukemia include hepatic infiltration with mature and immature cells of myeloid series. In lymphoid leukemia, the infiltrates consist of mature and immature cells of lymphatic series.^20^ Syncytial giant cell hepatitis,^16^ SOS, Budd-Chiari syndrome,^20^ ischemic hepatitis,^17^ sepsis,^18^ and cholestasis induced by total parenteral nutrition^19^ are other causes of hepatic dysfunction in patients with leukemia. We did not find any literature about the incidence and predictor factor for development of acute hepatitis in patients with leukemia. In our study, we observed acute hepatitis in one-third (34.6%) of patients with leukemia. Pattern of liver injury was mostly hepatocellular. Clinical course was favorable and we did not observe acute liver failure in any patients. Age (p = 0.001), HBsAg (p = 0.007), acute leukemia (p < 0.001), positivity of blood culture (p = 0.004), the amount of transfused red blood cell (p = 0.001), and amount of transfused platelets (p = 0.002) were significantly different under univariate analysis between the acute hepatitis group and the nonacute hepatitis group. But, under multivariate analysis, only ALL was identified as independent predictive factor for development of acute hepatitis after starting chemotherapy. The relationship between ALL and development of acute hepatitis may be related to treatment modality (i.e., prednisone/ methotrexate, etc.) or intensity of chemotherapeutic agent that using for treatment of ALL, or may be related to extensive replacement of hepatocytes with lymphoblast.

Drug-induced liver injury (DILI) is defined as any liver injury that is caused by medication. It may be dose-dependent, as seen in medication, such as aspirin and acetaminophen, or idiosyncratic.^11^ Drug-induced liver injury is the second main cause of acute liver failure and predominates in much of the developed world.^21^ In a study of postliver transplant patients, antibiotics were the most commonly implicated agents, although 14% of reported cases were due to immunosuppressive agents, including tacrolimus and azathioprine. Other studies confirm nonsteroidal anti-inflammatory drugs and antibiotics, especially amoxicillin-clavulanic acid as the most common causes of DILI. The clinical presentation of hepatic dysfunction may range from asymptomatic elevation of AST and ALT to fulminant hepatic failure. Liver injury can be described as cholestasis, hepatocellular, or mixed in nature.^11^ Hepatocellular injury is the more commonly occurring presentation and has been correlated with worse outcome. Other clinical manifestations include SOS and steatohepatitis.^11,22,23^ There are several criteria in use for diagnosing DILI and, although none are considered a gold standard, they can be used as tools to assist in the clinical diagnosis of DILI.^11^ Imatinib mesylate,^6,7^ hydroxyurea,^8^ L-asparaginase,^9^ arsenic trioxide,^10^ which are used in the treatment of leukemia, can cause hepatitis. High-dose chemotherapy alone or combined with total body irradiation, mainly cyclophosphamide in combination with either busulfan or total body irradiation or regimens that include carmustine, or multiple alkylating agents can cause SOS.^20^ None of our patients received total body irradiation. There were no findings compatible with SOS in patients with acute hepatitis. Our patients received many drugs, including several chemotherapeutic agents, antibiotics, immunosuppressive, and in a few patients, nonsteroidal anti-inflammatory drugs that can cause acute hepatitis. Additionally, septicemia and total parenteral nutrition can cause transient liver enzyme elevation. Therefore, we could not identify exact incidence and reasons of DILI in our patients.

Reactivation of hepatitis B infection is an important cause of acute hepatitis in patients with hematological malignancy. The risk of reactivation is higher with regimens containing rituximab or combination of rituximab and steroids. Chemotherapy-induced immune response impairment allows virus replication, and the restoration of immune response after treatment results in rapid destruction of infected hepatocytes. Hepatitis B virus reactivation ranges clinically from transient and silent to potentially severe HBV infection, resulting in acute life-threatening liver failure, particularly if immune suppression is continued and specific anti-HBV therapy is delayed.^10,24-26^ Available data suggest that prophylaxis is better than delaying treatment until serological evidence of reactivation is detected. Thus, all HBsAg-positive patients who undergo chemotherapy should receive prophylaxis. Although the largest body of evidence is available for lamivudine, there is a risk of resistance with this drug and newer analogs, such as tenofovir and entecavir are preferred.^26^ In our patients, there were 7 patients with chronic HBV infection before commencement of chemotherapy. Six out of these 7 patients had experienced acute hepatitis despite preemptive lamivudine treatment.

The HBV-DNA level did not elevate during development of acute hepatitis in these 6 patients. This finding was not compatible with classical feature of chemotherapy-induced acute exacerbation of chronic HBV infection. In univariate analysis, acute hepatitis was significantly higher in patients with HBV infection compared with patients without HBV infection after commencement of chemotherapy (p = 0.007). But in multivariate analysis, we did not find statistically significant correlation between HBV infection and the development of acute hepatitis. The number of our patients with HBsAg positivity was small. We suggest that the effect of preemptive lamivudine treatment in patients with leukemia should be investigated in larger and controlled studies.

In conclusion, acute hepatitis develops in important proportion of patients with leukemia. But, acute hepatitis recovers by conservative care and does not cause acute liver failure. The most important factor for development of acute hepatitis is the type of leukemia. The pathogenesis of leukemia-related acute hepatitis may be related to extensive malignant infiltration of liver tissue or may be related to drugs that are used for treatment.
